# Integrating Video and Objective Performance Data: Development of VORTEX-SPV for Assessing Robotic Lobectomy Skills

**DOI:** 10.1177/15569845251407699

**Published:** 2026-02-19

**Authors:** Alyssa D. Murillo, Riley Brian, Camilla Gomes, Laleh Foroutani, Amir Ashraf Ganjouei, Hueylan Chern, Patricia S. O’Sullivan, Daniel S. Oh

**Affiliations:** 1 Department of Surgery, University of California San Francisco (UCSF), CA, USA; 2 Department of Surgery, University of Southern California, Los Angeles, CA, USA

**Keywords:** assessment, objective performance indicators, robotic surgery, robotic surgery education, resident and fellow assessment, competency-based education

## Abstract

**Objective:**

Surgical education requires assessment tools as part of competency-based education (CBE). In robotic surgery, objective performance indicators (OPIs) are a novel objective method of measuring surgeon performance. We developed **V**ideo and **O**bjective **R**obotic **T**ask **E**valuation inde**X** on **S**uperior **P**ulmonary **V**ein dissection (VORTEX-SPV), combining video review and OPIs, to assess cardiothoracic (CT) fellow competency on SPV dissection during lobectomy.

**Methods:**

CT fellows performed robotic lobectomy on an ex vivo perfused porcine model. Synchronized video and kinematic data were captured. Seven thoracic surgery attendings completed the robotic lobectomy to provide expert-level OPI data. VORTEX-SPV is a 12-item assessment with 4 performance levels including completion, safety, economy of motion, and optimized performance. We scored video-based review items (items 1 to 6) as 0 or 1 with an inter-rater agreement of 91%. We calculated OPIs from 6 categories (e.g., wrist articulation, instrument movement, energy, smoothness, clutching, instrument time) as within (1) or beyond (0) 2 standard deviations of the expert mean. We used Rasch modeling and item response theory to evaluate reliability and validity.

**Results:**

We assessed 2 cohorts of CT fellows (2019, *n* = 50; 2023, *n* = 59). There were 60% (65 of 109) who had complete data. Reliability was high (expected a posteriori = 0.90). Fourteen trainees scored up to the level of completion, 10 at safety, 17 at economy of motion, and 24 achieved optimized performance. All items appropriately increased in difficulty (Spearman’s rho coefficient = 0.93).

**Conclusions:**

VORTEX-SPV combines video review and objective performance data with strong reliability and validity evidence, serving as a robotic surgery assessment framework to facilitate CBE.

## Graphical abstract

**Graphical Abstract:**
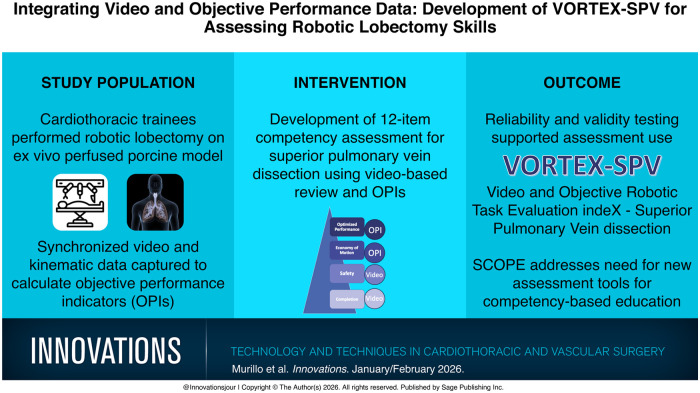
This is a visual representation of the abstract.

**Central Message**

VORTEX-SPV is a novel robotic surgery assessment with strong reliability and validity evidence, integrating video review with OPIs to evaluate robotic surgery competency. It supports the shift to competency-based training by enabling faculty to deliver objective, formative feedback that promotes resident development.

## Introduction

Surgical training has traditionally followed a time-based model, assessing trainees based on years of training and case numbers.^1^ However, advancements in surgical technology combined with reduced resident work hours have raised concerns about readiness for unsupervised practice.^2–5^ As a result, surgical training is transitioning to competency-based education (CBE), which emphasizes that all trainees must demonstrate required competencies before advancing.^6–9^ Cardiothoracic surgery programs in Canada have already implemented CBE, and U.S. programs are expected to follow within the next year.^10^ However, the primary assessment framework, Entrustable Professional Activities (EPAs), are not specific to surgical modality and do not assess technical skills specific to robot-assisted surgery (RAS).^9^ To operationalize robotic competency determination, novel assessment tools are needed.^11,12^

Several competency assessment tools exist with validity evidence for RAS, but most are judgment based and not specialty specific.^13–15^ Global Evaluative Assessment of Robotic Skills (GEARS) and Objective Structured Assessment of Technical Skills (OSATS) rely on observational assessment and do not leverage robotic kinematic data or system events.^14–16^ In GEARS, for example, the rater is asked to judge if the skills are “optimal,” “organized,” or “reasonable,” which can lead to variability between reviewers.^14^ Leveraging data from the robotic platform could improve the quality of assessment tools.

A unique aspect of RAS is that the movements and activities of the surgeon are translated into digital data, indicating aspects of surgical tasks such as dissection, retraction, and suturing. Objective performance indicators (OPIs) are metrics calculated from robotic kinematic and system data.^17^ OPIs provide quantitative, individualized performance data, which can provide feedback not available with laparoscopic or open surgery. Studies provide validity evidence for OPIs demonstrating the ability to distinguish between attendings and trainees as well as identify those prone to cause bleeding.^18–20^ Personalized performance data could support more reliable competency assessments and enhance formative feedback needed for CBE.

A novel application of the OPI data is to integrate it with video data, which reflects the traditional observational assessment. Such an approach we named “Video and Objective Robotic Task Evaluation indeX” (VORTEX). VORTEX is grounded in theories of skill acquisition and psychomotor learning as described under construct development. This article aims to model this approach by determining validity evidence for an assessment tool specific to superior pulmonary vein (SPV) dissection, called VORTEX-SPV. VORTEX-SPV combines observational assessment with OPIs from robotic system data specific to SPV dissection. Although this assessment is specific to SPV dissection on a perfused porcine model, we propose it as a framework for future assessments that can be adapted to other tasks, procedures, or models across robotic surgery training.

## Methods

### Assessment Tool Construct Development

We developed VORTEX-SPV using the BEAR Assessment System (BAS), a construct modeling approach that used 4 building blocks: construct map, item design, outcome space, and the calibration model.^21^ The construct map, item design, and outcome space are integral to assessment tool development, whereas the calibration model provides instrument and item validity evidence. The construct map is an ordinal and latent (unobservable) construct, with levels representing qualitative points of differentiation. Our construct was competency on the SPV dissection of the left lobectomy on a perfused porcine model. The task we selected was grounded in theories of psychomotor skill development and meets all 4 conditions of expertise development through deliberate practice: (1) given a task with a well-defined goal, (2) motivated to improve, (3) provided with feedback, and (4) provided with ample opportunities for repetition and gradual refinements of their performance.^22–25^ By leveraging objective performance data, we aimed to enhance formative assessment and, namely, the third condition of deliberate practice through targeted, learner-specific feedback, facilitating skill improvement.^24,25^

We focused on vein dissection rather than the entire lobectomy for multiple reasons. First, the skills involved in vein dissection are transferable to other types of vessel exposure in lobectomies and various surgical procedures. Second, OPIs are task dependent, making case segmentation essential for enhancing specificity.^18–20^ Third, it increased the likelihood of completion by all participants as it is the first step of the lobectomy in this context. Although we recognize that vein dissection is not always the first step in lobectomy, the instructional video for this lab and faculty instructed participants to begin with it. Finally, a prior publication demonstrated that performance during this initial step is associated with complications of bleeding at any point during the procedure.^18^

We selected an animal-based ex vivo model for its realistic simulation of human anatomy, allowing trainees to gain exposure and practice on the robotic console in a low-stakes environment. This model was perfused and pulsatile, adding to its realism. Furthermore, using a standardized model for a left upper lobectomy minimized variability compared with human subjects, which can affect the consistency and reliability of OPIs. Finally, this Advanced Tissue Model (ATM; Intuitive Surgical, Sunnyvale, CA, USA) is the same model used in robotic thoracic surgery training courses worldwide, presenting an opportunity for scaling the applicability of VORTEX-SPV in various training environments.^26^

**Fig. 1.**
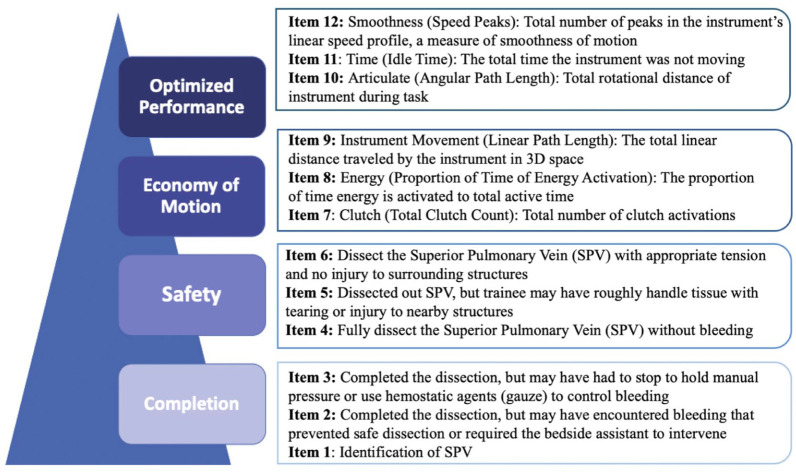
VORTEX-SPV is a novel assessment tool measuring trainee competency on superior pulmonary vein dissection on a perfused porcine model. Items 1 to 6 are video-based review items and items 7 to 12 are OPI items. OPI, objective performance indicator; VORTEX-SPV, Video and Objective Robotic Task Evaluation indeX on Superior Pulmonary Vein dissection.

To develop the qualitative levels of differentiation along the construct, we used prior literature on learning curves and the expertise of our team including thoracic surgeons from multiple institutions, surgical educators from our institution, data analysts, and surgeon educators from Intuitive Surgical.^27–29^ The team met virtually and adapted prior literature on learning curves, refining the 3 levels—development, competency, and mastery—by defining development as completion of the task, competency as safety, and mastery based on OPI metrics.^26^ This concept informed our construct map and defined skill in SPV dissection in a progressive, 4-level hierarchy that was composed of completion, safety, economy of motion, and optimized performance (Fig. 1). Completion is the base on the construct map as it reflects knowledge of the anatomy and terminal objective of the operative step, in this case, identification of the SPV and circumferential dissection of it to prepare for stapled ligation. In a human model, we expect lower completion rates compared with the porcine model because supervising surgeons would prevent trainees from persisting in unsafe scenarios. Safety is defined by completion of the operative step without evidence of bleeding or injury to surrounding tissues due to poor technique but also optimal exposure for a safe view of the target anatomy. Economy of motion, the third level, includes OPIs related to energy usage (cautery), finger clutching for repositioning, and instrument movement distance. In this hierarchy, economy of motion emphasizes the importance of minimizing unnecessary movements of the instruments, as efficient energy usage and clutching via the button and pedal activations on the console. It also reflects efficient task choreography so that the operative step is conducted without wasted time. Optimized performance, the final level, includes OPIs measuring wrist articulation, smoothness of hand movements, and instrument active time. These OPI metrics quantify how well a surgeon’s actions align with the principles of precision and control, which are the foundation of optimized performance.

### Assessment Tool Item Design

Figure 1 displays our assessment tool, VORTEX-SPV, which consists of 12 items with 3 assigned to each competency level (Fig. 1). Assessors manually scored items 1 to 6 using endoscopic video review, whereas items 7 to 12 were based on OPI data from the da Vinci data recorder (Intuitive Surgical). By including both video-based and system data-based OPI items, each group of metrics provides complementary context that the other alone cannot capture. For example, OPI metrics alone would not indicate whether bleeding or injury occurred. All items were scored dichotomously, and the grading rubric defined the outcome space.^21^

#### Video-based review items (items 1 to 6)

We developed the video-based review items by analyzing previously validated assessment tools of surgical trainees such as GEARS and OSATS.^13–15^ We finalized video-based review items through multidisciplinary discussion with surgical educators from our institution and Intuitive Surgical. Items 1 to 3 assess task completion, with bleeding as a possible reason for failure to complete the task. Items 4 to 6 correspond to safety and focus on the trainee’s incidence of bleeding, tissue handling, and injury to surrounding structures. The video-based items represented a patient safety concern, so if the trainee made any of these errors, then they received a 0 on that item and further scoring stopped, with the participants receiving an automatic 0 for any remaining items. Items 1 to 6 served as a gateway for assessing trainees on OPI data.

#### OPI selection and development of expert reference point for OPI items

For items 7 to 12, corresponding to the top 2 competency levels (economy of motion and optimized performance), we used OPI data. OPIs were broadly grouped into 6 categories: energy usage, console events, instrument movement, instrument time, smoothness, and wrist articulation. Each of items 7 to 12 correspond to these 6 OPI categories. We selected individual OPIs based on prior literature across various procedures and specialties.^18–20,30–36^ The final OPI selection is listed in Table 1.

**Table 1.** List of OPI Categories and Their Respective OPI Metrics.

OPI category	OPI	Description
Smoothness	Speed peaks	Total number of peaks in the instrument’s linear speed profile, a measure of smoothness of motion
Wrist articulation	Angular path length	Total rotational distance of instrument during task
Instrument movement	Linear path length	Total linear distance traveled by the instrument in 3D space
Instrument active time	Idle time	Total time the instrument was not moving
Energy usage	Proportion of time energy was activated to total active time	Amount of time energy is activated
Clutching	Number of clutch activations	Total clutch count

Abbreviations: 3D, 3-dimensional; OPI, objective performance indicator.

OPI metrics selected based on principal component analysis to determine OPIs most predictive for identifying differences between expert and novice in the 6 OPI categories.

### Data Collection

We collected data during The Society of Thoracic Surgeons (STS) cardiothoracic “Boot Camp” in 2019 and 2023. First-year cardiothoracic fellows from across the United States participate in this program to acquire foundational skills in core procedures such as robotic lobectomy. Each of the cardiothoracic surgical residents received 2 h of supervised experience performing a left upper robotic lobectomy on a perfused porcine tissue simulator (ATM). Prior to the robotic lobectomy, participants provided informed consent to participate and completed demographic information and their prior robotic simulation and console experience. The data from 2020 to 2022 were not available due to the COVID pandemic. We reported descriptive summaries as frequencies and percentages for categorical variables and medians and ranges for continuous variables. We performed no group comparisons because that was not relevant to the validity evidence analysis.

During the lobectomy, data were collected through a recorder connected to the da Vinci Xi surgical system (Intuitive Surgical) that simultaneously captured video (60 frames per second) as well as kinematic (i.e., instrument movements collected at 50 Hz) and event data (i.e., button presses on the system such as camera clutch and energy application). All participants had unique identifiers.

### Expert OPI Norm for Trainee Performance

During the STS Boot Camp, 7 academic thoracic surgeon attendings from across the United States also completed the SPV dissection without bleeding or injury to the vessel. We calculated the mean and standard deviation for each OPI in the 6 categories. After examining the histograms comparing trainees and experts for each OPI, we set the trainee credit threshold at 2 standard deviations within the mean of the attendings. Trainees with OPI values outside this range did not receive credit for that item. Although this cutoff point was based on data analysis, we acknowledge that it represents 1 criterion, and other criteria are also possible.

### Calibration Model: Reliability and Validity Testing

The final step of the BAS system is the calibration model, composed of multifaceted reliability and validity testing of VORTEX-SPV. For reliability testing, we calculated inter-rater reliability for the video-based items and item response theory for the instrument itself. Two independent raters (A.M., C.G.) retrospectively reviewed the endoscope images to evaluate trainee performance. Inter-rater agreement was high at 91%, with a Kappa of 0.84, indicating that the items enabled clear decisions and resulted in substantial agreement between 2 independent raters. Finally, to test the instrument itself, we applied item response theory, with expected a posteriori (EAP) and weighted likelihood estimation (WLE) methods, to assess the true ability of the trainee and how the trainee was performing on the instrument.

For validity evidence, we employed several methods including internal structure evidence, content validity, and fairness evidence. We examined the internal structure validity through Rasch modeling to generate fit statistics, mean respondent and item response threshold locations, and a Wright Map.^21,37^ Fit statistics refers to how well an individual assessment item conforms to the expectations of the Rasch measurement model. It indicates whether the item behaves predictably across the range of participant abilities. We used a range of 0.7 to 1.3 as indicative of a good fit.^37^ The video-based items are expected to have low infit given that they are gated and not uniform across participants. Mean person ability refers to the estimated level of the latent trait (e.g., competency on SPV dissection) for each participant, whereas item location reflects the difficulty level of each assessment item on the same logit scale. To provide instrument internal evidence, we calculated a biserial correlation between item location and the mean person ability at each performance level to assess whether item scores increased in a manner consistent with increasing ability.

The Wright Map visually positions mean respondent locations and item response thresholds along the construct, using a logit scale. We analyzed the Wright Map by evaluating its alignment with the proposed construct and the presence of threshold banding. Spearman’s rho coefficient assessed the strength and direction of the relationship between the theoretical construct map and the empirical Wright Map.

We gathered fairness evidence by conducting subgroup analyses, comparing male with female trainee performance, to ensure that the tool was equitable across diverse trainee groups. We assessed fairness by calculating the correlation between mean person ability estimates for female and male participants across the items. A high correlation suggests that items function similarly across groups, indicating fairness in measurement. We also compared the confidence intervals of mean person ability estimates for each item; substantial overlap between groups was interpreted as evidence of no meaningful difference in item performance by sex. The fairness evidence analysis included only the 2023 cohort, which had demographic data matched to the OPI data.

### Application of VORTEX-SPV Assessment Tool

OPI metrics are task and model specific; therefore, VORTEX-SPV is limited to the assessment of the SPV dissection on the ATM perfused porcine model. As such, it is not directly generalizable to other steps of an upper lobectomy or to human patients without recalculating expert performance benchmarks and conducting the same rigorous reliability and validity testing to validate the assessment. Although our assessment is currently specific to a single model and task, VORTEX-SPV offers a foundational assessment framework that could be adapted to other tasks and training models in the future.

## Results

We included 109 cardiothoracic trainees from the 2019 (*n* = 50) and 2023 (*n* = 59) STS Boot Camps. Prior console simulation experience was common in both cohorts. However, the 2023 trainees had more simulation exposure. Clinical robotic console experience was also higher in 2023. None of the trainees had prior experience with the lobectomy model (Table 2).

**Table 2.** Descriptive Results of Both the 2019 and 2023 Cohorts.

	2019 cohort		2023 cohort	
	Number (%)	Mean (range)	Number (%)	Mean (range)
Number of fellows	50		59	
Integrated I-6 track	7 (14%)		8 (14%)	
Traditional cardiothoracic track	42 (86% of reported)		51 (86%)	
Experience with robotic simulation	48 (100% of reported)	7 (0.5–30) h	49 (83%)	17 (2–50) h
Robotic console experience	40 (82% of reported)	22 (1–63) h	54 (92%)	33 (2–150) h
Completed data for analysis	35 (70%)		30 (51%)	
Completed superior pulmonary vein dissection without bleeding or injury	21 (60%)		22 (73%)	
Bleeding event	8 (23%)		7 (23%)	
Demonstrated poor tissue handling	4 (11%)		0	
Did not maintain tension during dissection	2 (6%)		1 (3%)	

Both cohorts demonstrated more trainees from traditional fellowship tracks compared with integrated programs. More trainees had simulation experience in 2019, but the 2023 cohort had more hours completed. The 2019 cohort had similar console experience to the 2023 cohort, with fewer hours. A total of 65 trainees (60%) had complete data and were included in the analysis. Both cohorts had a similar number of bleeding events, with more trainees in the 2019 cohort causing tissue injury or not maintaining tension.

For the remaining analysis, we included only the 65 participants (35 from 2019 and 30 from 2023) with complete data. We excluded 44 participants lacking full data from the recorder, which failed periodically and randomly amongst participants during the study period.

**Fig. 2.**
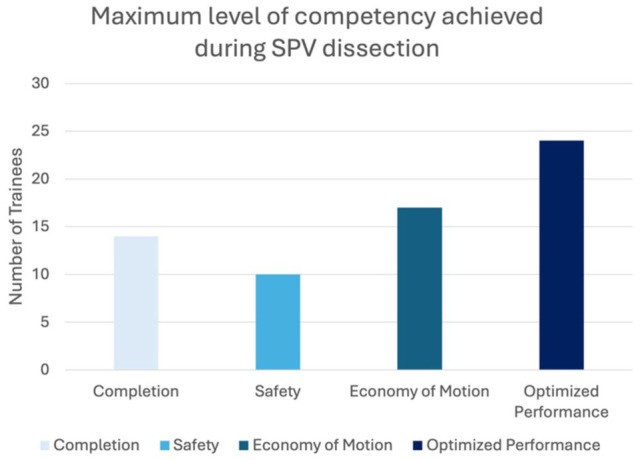
The trainee scores on VORTEX-SPV. Most trainees scored in optimized performance, although trainees were represented at all levels of competency. VORTEX-SPV, Video and Objective Robotic Task Evaluation indeX on Superior Pulmonary Vein dissection.

Of the 65 participants, 15 trainees (23.1%) experienced bleeding during their dissection and therefore received a score of 0 on items 2, 3, or 4 (Table 2). Seven additional trainees (10.8%) demonstrated poor tissue handling (item 5) or did not maintain tension during their dissection (item 6). These 22 trainees received a score of 0 for all remaining items. The remaining 66.2% of trainees (43 of 65) received credit for all the video-based items and were consequently able to receive scores on the OPI items based on the graded assessment tool. Although most trainees achieved optimized performance, individuals were represented across all competency levels (Fig. 2).

### Reliability Testing

The EAP reliability coefficient for the assessment tool was 0.903, and the WLE reliability coefficient was 0.878, indicating a high level of internal consistency of the assessment tool within the context of item response theory.

### Validity

#### Item and instrument internal structure

**Fig. 3.**
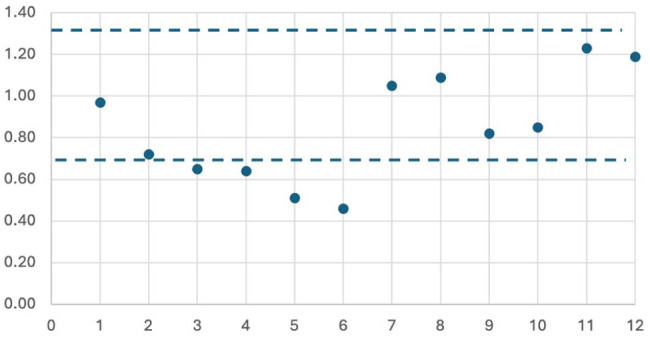
Analysis of item fit by number with a cutoff of 0.75 and 1.33 for item performance. Items 2 to 6 demonstrated that they were under the 0.75 threshold, likely due to nonuniformity from gated items. All other items were within ideal range.

Items 1 and 7 to 12 demonstrated good fit. Items 2 to 6 showed low fit due to nonuniformity, resulting from the automatic score of 0 assigned to all remaining items when a 0 was given on a video-based item (Fig. 3). Biserial correlation with the mean person ability and item location demonstrated items appropriately increased across the 4 levels (Supplemental Table 1). Both findings support the constructs’ ordinal nature and provide internal structure validity evidence.

**Fig. 4.**
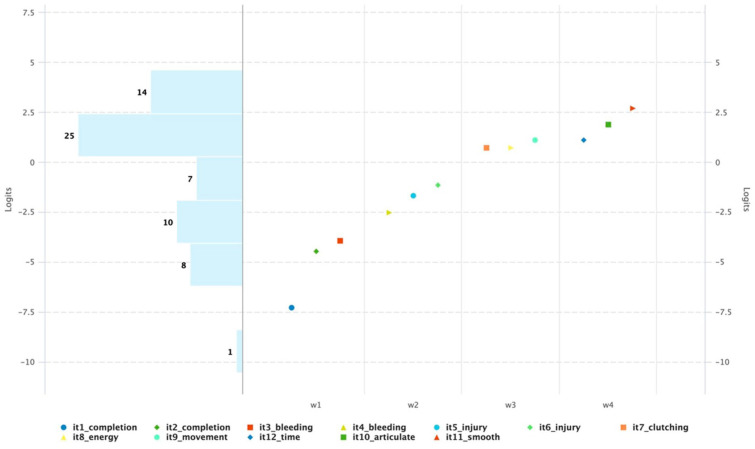
Wright Map for measuring trainee competency on robotic porcine superior pulmonary vein exposure. The levels are represented as follows: W1 = completion, W2 = safety, W3 = economy of motion, and W4 = optimized performance. Items are listed in the legend. Items increase in difficulty across levels with banding between each level, supporting an ordinal construct with items functioning as expected.

The Wright Map demonstrated a positive linear relationship with the theoretical construct map, confirming that each waypoint occurs sequentially with increasing difficulty as predicted (Fig. 4). There is threshold banding across the instrument with items occurring in the correct level, which suggest that the items in each level are within a similar range of difficulty. The Spearman’s rho coefficient was 0.93, also suggesting the ordering of difficulty levels in the empirical data aligned well with the expected progression of competency levels in the construct map.

In addition, the alignment between the empirical item difficulty and the theoretical construct provides content validity evidence. Trainee performance on VORTEX-SPV aligned with the expected skill progression outlined in the construct map, suggesting that the theoretical framework effectively captured the essential elements of competency on SPV dissection.

#### Fairness testing

**Fig. 5.**
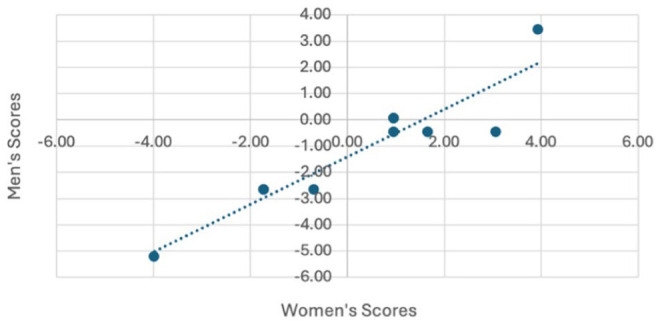
Fairness validity testing with item difficulty by self-reported sex of trainees with a correlation of 0.93 supporting no difference in performance on the assessment by sex.

We assessed fairness by comparing item performance between male and female trainees in the 2023 cohort (13 female and 17 male trainees). Due to identical scores on the first 2 items among male trainees, the analysis focused on items 3 to 12. The strong correlation (*r* = 0.93) between male and female trainee scores indicates no sex-related bias (Fig. 5). Overlapping confidence intervals in mean person ability scores further support fairness (Supplemental Table 2). These findings confirm that VORTEX-SPV provides an equitable assessment, with no statistical evidence of sex-related bias.

## Discussion

We created VORTEX-SPV, a novel assessment tool to measure trainee competency during the SPV dissection of a left upper lobectomy on a perfused porcine model that incorporated both video-based review and objective performance data. The assessment tool demonstrated high reliability supported by high inter-rater reliability on the video-based items and principles of item response theory. We conducted a comprehensive validity assessment, examining multiple facets including content analysis, internal structure of the instrument and items, and fairness. Validity testing consistently supported the accuracy and effectiveness of the instrument. Although VORTEX-SPV is specific to both the task and the model due to the nature of the OPIs, we present it as a foundational framework with the potential to be adapted to other tasks, procedures, or models.

We believe VORTEX-SPV provides trainees with a formative assessment that they could use to engage in deliberate practice, addressing the reported lack of feedback.^11–15,22–25,38–40^ VORTEX-SPV raises the quality of feedback trainees receive with both video-based review and OPI metrics.^30,31^ The OPIs from the robotic platform, which are now commercially available, offer an unprecedented level of individualized performance feedback with a level of specificity that could not be captured through observation alone. This presents an opportunity to design assessments using the VORTEX approach by incorporating standardized performance metrics generated by the robotic system.^14–16^ This could also address the need for innovative and effective assessment tools for competency-based assessment in robotic surgical education.

One consideration is the retrospective nature of our study. We completed the first half of the assessment with a retrospective video review, and the OPI metrics required processing, necessitating time for annotating the videos and analyzing the data from the SPV dissection. In the future, there is the possibility for immediate delivery of the assessment following case completion. The attending could complete the first 6 items of the formative assessment at the end of a procedure as a micro-assessment. The OPI metrics could also be immediately available following an operation through automated case segmentation to identify the SPV step and edge-based calculations of OPIs. Therefore, both forms of the assessment could be combined for immediate feedback in the form of the trainee scores or their performance in comparison with their peers. Immediate feedback delivery to trainees would allow for reinforcement of learning with real-time guidance, enabling them to make immediate corrections and adjustments. In addition, advancements in machine learning create the potential for automating the OPI score of this assessment, reducing the feedback burden on supervising surgeons.

A limitation of our study is that VORTEX-SPV is designed for only SPV dissection in a porcine model and therefore may not be applicable on a different task or procedure. The OPIs are highly specific to the task, making the expert OPI cutoff scores not transferable to other tasks. This selection prototypes developing an assessment with reliability and validity evidence while anticipating a broader application to other procedures. Of note, the same perfused porcine model is currently used in the Intuitive Surgical thoracic surgery training course; therefore, there is an opportunity to apply VORTEX-SPV across multiple training environments.^26^ Future efforts should focus on developing additional task-specific assessments or expanding the collection of validity evidence for this tool to assess vessel exposure and dissection in other types of operations or models.

## Conclusions

We demonstrated strong validity and reliability evidence for VORTEX-SPV in assessing SPV dissection skills, reinforcing its potential as a valuable tool for formative assessment and structured feedback. By integrating video-based review with OPIs, VORTEX-SPV enhances the reliability and consistency of trainee assessments, addressing long-standing challenges associated with purely judgment-based assessments. VORTEX-SPV represents a significant advancement in competency-based assessment for robotic thoracic surgery training and addresses the need for assessments to operationalize CBE.

## Supplemental Material

sj-docx-1-inv-10.1177_15569845251407699 – Supplemental material for Integrating Video and Objective Performance Data: Development of VORTEX-SPV for Assessing Robotic Lobectomy Skills Supplemental material, sj-docx-1-inv-10.1177_15569845251407699 for Integrating Video and Objective Performance Data: Development of VORTEX-SPV for Assessing Robotic Lobectomy Skills by Alyssa D. Murillo, Riley Brian, Camilla Gomes, Laleh Foroutani, Amir Ashraf Ganjouei, Hueylan Chern, Patricia S. O’Sullivan and Daniel S. Oh in Innovations
