# Supplementary material for: Integrating Video and Objective Performance Data: Development of VORTEX-SPV for Assessing Robotic Lobectomy Skills
Source: Innovations (Phila). 2026 Feb 19;21(1):64–72. doi: 10.1177/15569845251407699 (PMC13049217; doi:10.1177/15569845251407699)
Supplement: sj-docx-1-inv-10.1177_15569845251407699 – Supplemental material for Integrating Video and Objective Performance Data: Development of VORTEX-SPV for Assessing Robotic Lobectomy Skills [file sj-docx-1-inv-10.1177_15569845251407699.docx]

**Supplemental Table 1.** Biserial Correlation With the Mean Person Ability Demonstrating All Items Were Performing as Expected.

| **Item number** | **Item** | ***N*** | **Cat** | **Number of respondents** | **Proportion of respondents** | ***r_pb_* WLE** | **M WLE** |
| --- | --- | --- | --- | --- | --- | --- | --- |
| 1 | it1_completion | 65 | 0 | 1 | 0.02 | -0.34 | -8.39 |
| 1 | it1_completion | 65 | 1 | 64 | 0.98 | 0.34 | 0.14 |
| 2 | it2_completion | 65 | 0 | 7 | 0.11 | -0.68 | -6.09 |
| 2 | it2_completion | 65 | 1 | 58 | 0.89 | 0.68 | 0.75 |
| 3 | it3_bleeding | 65 | 0 | 9 | 0.14 | -0.74 | -5.71 |
| 3 | it3_bleeding | 65 | 1 | 56 | 0.86 | 0.74 | 0.93 |
| 4 | it4_bleeding | 65 | 0 | 15 | 0.23 | -0.82 | -4.67 |
| 4 | it4_bleeding | 65 | 1 | 50 | 0.77 | 0.82 | 1.42 |
| 5 | it5_injury | 65 | 0 | 19 | 0.29 | -0.86 | -4.17 |
| 5 | it5_injury | 65 | 1 | 46 | 0.71 | 0.86 | 1.74 |
| 6 | it6_injury | 65 | 0 | 22 | 0.34 | -0.87 | -3.79 |
| 6 | it6_injury | 65 | 1 | 43 | 0.66 | 0.87 | 1.96 |
| 7 | it7_clutching | 65 | 0 | 34 | 0.52 | -0.71 | -2.10 |
| 7 | it7_clutching | 65 | 1 | 31 | 0.48 | 0.71 | 2.33 |
| 8 | it8_energy | 65 | 0 | 34 | 0.52 | -0.70 | -2.08 |
| 8 | it8_energy | 65 | 1 | 31 | 0.48 | 0.70 | 2.31 |
| 9 | it9_movement | 65 | 0 | 37 | 0.57 | -0.73 | -1.95 |
| 9 | it9_movement | 65 | 1 | 28 | 0.43 | 0.73 | 2.61 |
| 10 | it10_articulate | 65 | 0 | 43 | 0.66 | -0.66 | -1.45 |
| 10 | it10_articulate | 65 | 1 | 22 | 0.34 | 0.66 | 2.88 |
| 11 | it11_smooth | 65 | 0 | 49 | 0.75 | -0.44 | -0.78 |
| 11 | it11_smooth | 65 | 1 | 16 | 0.25 | 0.44 | 2.43 |
| 12 | it12_time | 65 | 0 | 37 | 0.57 | -0.66 | -1.77 |
| 12 | it12_time | 65 | 1 | 28 | 0.43 | 0.66 | 2.37 |

Abbreviation: WLE, weighted likelihood estimation.

**Supplemental Table 2.** Fairness Evaluation With Confidence Intervals for Performance of Men and Women on Each Item.

| **Scoring guide** | **Women** | **Men** | ***P* value** |
| --- | --- | --- | --- |
| it3_bleeding | -3.98 (-11.78, -6.13) | -5.22 (-15.45, -7.77) | >0.05 |
| it4_bleeding | -1.72 (-5.09, -3.76) | -2.65 (-7.84, -4.65) | >0.05 |
| it5_injury | -1.72 (-5.09, -3.76) | -2.65 (-7.84, -4.65) | >0.05 |
| it6_injury | -0.69 (-3.76, 0.32) | -2.65 (-7.84, -4.65) | >0.05 |
| it7_clutching | 0.96 (-0.71, 2.63) | 0.05 (-1.28, 0.15) | >0.05 |
| it8_energy | 0.96 (-0.71, 2.63) | 0.05 (-1.28, 0.15) | >0.05 |
| it9_movement | 1.66 (0.03, 3.29) | -0.45 (-1.88, -1.33) | >0.05 |
| it10_articulate | 3.07 (1.35, 4.79) | -0.45 (-1.88, -1.33) | >0.05 |
| it11_smooth | 3.93 (2.01, 5.85) | 3.44 (1.73, 10.18) | >0.05 |
| it12_time | 0.96 (-0.71, 2.63) | -0.45 (-1.88, -1.33) | >0.05 |

Overlapping intervals suggest no difference in performance by sex. Items 1 and 2 excluded due to lack of variability in women.

All comparisons were not significant (*P* > 0.05).
